# Prevalence of Visual Impairment Among Students Before and During the COVID-19 Pandemic, Findings From 1,057,061 Individuals in Guangzhou, Southern China

**DOI:** 10.3389/fped.2021.813856

**Published:** 2022-02-11

**Authors:** Jing-hong Liang, Yi-can Chen, Yu Zhao, Aerziguli Kakaer, Nan Jiang, Shan Huang, Shu-xin Zhang, Ya-jun Chen

**Affiliations:** Department of Maternal and Child Health, School of Public Health, Sun Yat-sen University, Guangzhou, China

**Keywords:** COVID-19 pandemic, public health, students, successive cross-sectional study, visual impairment

## Abstract

**Background:**

Visual impairment (VI) is a growing public health concern among students as a result of the COVID-19 pandemic.

**Objective:**

This study investigated the change in VI before and during the pandemic among students.

**Methods:**

Data on 547,864 and 497,371 students were obtained from the Guangzhou Survey on Students' Constitution and Health (GSSCH) collected in October 2019 and October 2020, respectively. VI was defined as the unaided distance visual acuity lower than 20/25 Snellen equivalent (LogMAR 0.10) in the worse eye. Change in VI based on age and sociodemographic variables were evaluated by *chi-square* test for trend as appropriate. Comparisons of different categorical variables were tested by contingency tables-based *chi-square test*. We have further analysis of the students who went through both of the 2019 and 2020 examinations for evaluating the VI incidence during the COVID-19 pandemic.

**Results:**

More than 1,045,235 students were involved in our study, among whom 271,790 (54.65%) out of 497,371 students in Guangzhou suffered from VI during the COVID-19 compared with 293,001(53.48%) visually impaired students (total tested participants = 547,864) before the COVID-19 pandemic. During the pandemic, the overall prevalence of VI actually showed an increased age tendency and reached the highest level in the 17 [80.04%, 95%Confidence interval (CI):79.53 to 80.54%] and the 18 (79.64, 95%CI: 79.06 to 80.23%) age groups. Rapid growth was detected among students aged between 9 and 16 years old (raised by 46.21) while older students were more likely to get moderate and severe VI than younger ones. Students involved in more screen-based activities [(64.83%, 2019); (66.59%, 2020)] appeared to have a higher prevalence of VI than those involved in less [(49.17%, 2019); (49.26%, 2020)].

**Conclusion:**

A rising trend of VI among students was detected before and during the COVID-19 pandemic. Moreover, the pandemic appeared to be associated with a rapid VI shift in younger and boy populations. Potential danger may arise when public health emergencies occur in the school, and more effort should be made to improve students' vision.

## Introduction

Heated debates were carried out in both academic circles and public arenas respecting whether children and adolescents obtain the relative ability to acquire and spread severe acute respiratory syndrome coronavirus 2 (SARS-CoV-2), namely, the causative agent of coronavirus disease 2019 (COVID-19). Although it is well-established that the pandemic is more commonly susceptible in other age groups than children and adolescents, the relevant health outcomes affected by the virus still do not draw enough attention to it, visual impairment (VI) in particular. VI was considered as the considerable impact that posing a battery of adverse consequences of academic performance, career choices on students, the social life in particular (1, 2), and further impair the development of their physical and mental-related diseases such as diabetic retinopathy and cognitive function (3, 4).

The students performing their ability optimally to achieve various tasks that require the operation of peripheral vision will be influenced if they suffered from VI due to the estimate of 85% of students acquired their knowledge in school through visual presentation (1, 5, 6). Such problems have imposed a tremendous burden on students themselves, society, and even higher, creating a significant problem on the global healthcare system.

During the 2020 Spring Festival, the Chinese government took the emergency domestic quarantine measure of nationwide school closure to control the transmission of COVID-19, which lead more than 200 million students to be confined in their homes and to have to finish their studies delivered by the internet. Concerns about whether the duration and intensity of lockdown would overburden VI with the progressive decrease in outdoor spending and the direct increase in screen time have recently been raised (7). China's Ministry of Education issued an online survey comprised of 14,532 students around nine provinces in September 2020, which indicated that the myopic prevalence increased by 11.7% compared with the end of 2019 in China (8). Myopia is the most common cause of VI worldwide, which has developed into an urgent public health issue in east Asian countries, particularly in China where myopia is highly prevalent. Before and during the COVID-19 pandemic, changes in the incidence and demographic characteristics of VI among students have possibly appeared. The vast majority of VI is either preventable or treatable (9, 10).

Consequently, it is necessary, feasible, and even urgent now to document the prevalence of VI in school-age students and enhance the ability to handle emergent public health events in school to protect the students' vision. We then aimed at investigating the incidence of the VI among students before and during the COVID-19 pandemic by using the Guangzhou Survey on Students' Constitution and Health (GSSCH) collected on October 1, 2019 and 2020, respectively, with a view to providing novel proposals and strategy for application of COVID-19 based on the general feature of the changes in VI.

## Methods

### Patient and Public Involvement

The survey is not publicly available and participants were protected under a certificate of confidentiality issued by the Government of Guangzhou due to the sensitive nature of data collected from all students group in Guangzhou city. Requests to assess the dataset from qualified researchers trained in human participant confidentiality protocols may be sent to the School of Public Health, Medical College of Sun Yat-Sen University at chenyj68@mail.sysu.edu.cn.

The study was in accordance with the Strengthening the Reporting of Observational Studies in Epidemiology (STROBE) reporting guideline for cross-sectional studies (11). Written informed consent was obtained from all participants' parents or their legal guardians which was performed according to the guidelines of the Declaration of Helsinki (12). The studies involving human participants were reviewed and approved by the Ethics and Human Subject Committee of Sun Yat-Sen University. The participants provided their written informed consent to participate in this study.

Data were obtained from the GSSCH. A sequential cross-sectional investigation carried out annually was comprised of approximately 1,600 primary and middle schools in Guangzhou city, which is done and supported by Sun-Yat Sen University, Guangzhou school-health promotion center and Guangzhou Education Bureau. The host of relevant measures of GSSCG was done during September and October, the first 2 months of a new school year. The GSSCH in 2019 and 2020 academic year were performed between September and October in 2019 and 2020, respectively, and therefore defined as the period before COVID-19 and during the COVID-19 pandemic.

Data supporting our study is not publicly available due to the inherent sensitive nature of data. Therefore, to protect our study participants' privacy and ensure the data analyzed were independent, we randomly sampled half of the tested participants from the 1,286,244 and 1,067,983 available in 2019 and 2020, respectively, within each district. A data preprocessing was performed to eliminate the abnormal values and 131,879 students were excluded as follows: 105,503 were excluded because they did not explicitly report the key information on Uncorrected visual acuity (UVA), whether or not they did not on left or right eye. A total of 23,738 were removed because they were aged above 25, and a further 3,638 were excluded for missing the values on the other variables such as sex or grade.

### Visual Impairment Measurement

Students' eyesight was measured using the standard logarithmic visual acuity chart (the 5-mark record recommended by standardization administration of China), namely, retro illuminated logMAR chart with tumbling-E optotypes (Precision Vision) (13, 14) by detecting the UVA of their left and right eye separately. And the Visual Acuity (VA) of each student was converted into the logarithm of the minimum angle of resolution (LogMAR) value for all calculations. They were presenting LogMAR VA at a distance of 5 meters with tumling-E optotypes in a quiet and well-lit room. The suspension height of the chart should be ensured to be horizontal with the eyes of most subjects and its illumination was required between 300–500 lux. If the orientation of at least 4 of 5 optotypes on the LogMAR-1.0 (Snellen 6/60) line were correctly identified, students were re-examined on the LogMAR 0.70 (Snellen 6/30) line, the LogMAR 0.40 (Snellen 6/15) line, and the line by line to LogMAR-0.30 (Snellen 6/3). VA for an eye was defined correctly when the lowest line on which 4 of 5 optotypes were recorded (15).

The measurement of VA was performed by a selected group of medically trained health professionals (certified optometrist or ophthalmologist) who were trained by experienced attending ophthalmologists and the staff of attending physicians with active credentials. The training was taken to adhere to the specific and detailed protocols that was published by the Standardization Administration of China that use the same principles and technology over the years by all technicians. The test results were from each student in 3 trials. As defined according to the International Council of Ophthalmology in cooperation with the World Health Organization and the International Agency for the Prevention of Blindness (15, 16), with an UVA of both eyes above or equal to 20/20 Snellen equivalent was defined as normal vision while the UVA of any single eye below 20/25 Snellen equivalent (LogMAR 0.1) was defined as VI, which was classified into three groups, namely, mild, moderate, and severe VI. For judging VI, a mild VI rating was defined as UVA <20/25 Snellen equivalent and that of better or equal to 20/63 Snellen equivalent in the worse eye (i.e., logMAR vision <0.1 and ≥0.5), a moderate to severe VI rating was defined as UVA worse than 6/18 (20/63 Snellen equivalent, i.e., logMAR vision <0.5).

### Statistical Analyses

Data were collected and subsequently cleaned for invalid entries using Microsoft Excel (Microsoft Corp, Redmond, WA, USA). For categorical parameters (including socioeconomic and demographics information), the *Chi-square* test was done to compare them between groups. We have further analyzed the students who went through both the 2019 and 2020 examinations for evaluating the VI incidence during the COVID-19 pandemic. Four categories were calculated and defined, namely, 2019 without VI while 2020 with VI (Type 1), 2019 without VI while 2020 without VI (Type 2), 2019 with VI while 2020 without VI (Type 3), and 2019 with VI while 2020 with VI (Type 4).

The enumeration variables are expressed as counts with percent values. The Mantel-Haenszel *chi-square test* was used to perform two-group comparisons and statistical tests for trend, respectively. Furthermore, comparisons different categorical variables were tested by contingency tables-based *chi-square test*. Analyses were conducted with SAS software (Version 9.4, SAS Institute, Care, N.C), and STATA (version 14.0, Corp, College Station, TX.14.0), and two-tailed of *P* < 0.05 was considered statistically significant.

## Results

A total of 1,045,235 students (2,090,470 eyes) were included in our study, and 564,727 (54.29%) were boys. The total number of included students who accepted testing was 547,864 [50% of the 2019 sample size (1,286,244)] and 497,371 [50% of the 2020 sample size (1,067,983)] in 2019 and 2020, respectively, the former one with 293,664 boys (53.60%) and 254,200 girls (46.40%) while the other contained 271,063 boys (54.50%) and 226,308 girls (45.50%). The students in primary school, whether in 2019 or 2020, have been contributing the maximum weight on detected participants for our analysis (*N* = 358,861 in 2019, *N* = 323,413 in 2020), while the high school students got the minimum (*N* = 72,241 in 2019, *N* = 64,000 in 2020).

### Changes in Visual Impairment

The prevalence of VI in students before and during the pandemic, stratified by sociodemographic and behavioral factors, is shown in Table 1. Compared with lower-income households (monthly income <5,000 RMB), a higher prevalence of VI among middle-to-high income households (monthly income >5,000 RMB) was detected in both years (*P* < 0.001). Adolescents (aged 13–18 years) or high-school students had attained a greater prevalence of VI than children (aged 6–12 years) or primary and junior high school students in both years (*P* < 0.001). Of note, a relatively lower prevalence of VI was observed among students involved in more time on outdoor time while the students spending less time on outdoor time appear to have a higher prevalence of VI (*P* < 0.001). Similarly, VI prevalence was significantly higher among students involved in more time on the screen-based behavior than students involved in less time (*P* < 0.001). For the item of parental myopia, no statistically significant difference was found (*P* = 0.653). Supplementary Table 5 displays the baseline sociodemographic characteristics of involved students before and during the COVID-19 pandemic. The differences of the gender, age, socioeconomic status, and other movement time were statistically significant for 2 years while no statistical difference was observed for grade and parental myopia variables between 2019 and 2020.

**Table 1:** Prevalence of VI before and during COVID-19 pandemic among school-aged students stratified by sociodemographic factors.

**Parameter**	**Categories**	**Prevalence of visual impairment, No./Total No. (%)**		***P*-Value**
		**2019**	**2020**	
Gender	Boys	50,730/106,664 (47.56)	86,924/178,526 (48.69)	<0.001
	girls	51,818/93,343 (55.51)	86,318/153,100 (56.38)	
Age	Children	58,593/141,854 (41.31)	97,698/231,024 (42.29)	<0.001
	Adolescents	43,955/58,153 (75.59)	75,544/100,602 (75.09)	
Grade	Primary-school	45,127/115,032 (39.23)	83,577/207,443 (40.29)	<0.001
	Secondary-school	27,786/41,699 (66.63)	57,351/84,582 (67.81)	
	High-school	19,313/23,649 (81.67)	32,314/39,601 (81.60)	
Socioeconomic statues (monthly income/RMB)	<5,000	40,351/80,889 (49.88)	69,166/136,327 (50.74)	<0.001
	5,000–7,999	21,579/41,574 (51.91)	36,372/68,984 (52.73)	
	8,000–11,999	13,320/25,047 (53.18)	22,271/40,980 (54.35)	
	≥12,000	27,297/52,495 (52.00)	27,986/51,955 (53.87)	
Parental myopia	Yes	47,963/102,404 (46.84)	80,875/169,308 (47.77)	0.653
	No	54,584/97,601 (55.93)	92,367/162,318 (56.90)	
Total Outdoor time, h/d	T <1	43,580/82,625 (52.74)	78,086/147,247 (53.03)	<0.001
	1 ≤ T <2	42,764/84,612 (50.54)	70,510/135,906 (51.88)	
	T≥2	16,203/32,768 (49.45)	24,645/48,472 (50.84)	
Sunshine-related outdoor time, h/d	T <1	34,788/66,114 (52.62)	64,472/121,679 (52.99)	<0.001
	1 ≤ T <2	44,166/86,514 (51.05)	72,753/139,646 (52.10)	
	T≥2	23,593/47,377 (49.80)	36,016/70,300 (51.23)	
Total Screen-based time, h/d	T <1	60,464/122,962 (49.17)	97,227/197,373 (49.26)	<0.001
	1 ≤ T <2	26,378/50,705 (52.02)	46,087/86,803 (53.09)	
	2 ≤ T <3	7,811/12,924 (60.44)	16,656/27,168 (61.31)	
	3 ≤ T <4	2,788/4,251 (65.58)	6,239/9,473 (65.86)	
	T≥4	2,336/3,603 (64.83)	6,436/9,665 (66.59)	
Study-related screen-based time, h/d	T <1	3,617/7,084 (51.06)	7,461/14,420 (51.74)	<0.001
	1 ≤ T <2	24,178/53,294 (45.37)	41,762/88,627 (47.12)	
	2 ≤ T <3	37,343/75,448 (49.50)	62,211/122,683 (50.71)	
	3 ≤ T <4	24,496/43,217 (56.68)	40,686/71,692 (56.75)	
	T≥4	11,686/18,575 (62.91)	20,440/32,870 (62.18)	
Entertainment-related screen-based time, h/d	T <1	NA	130,917/254,462 (51.45)	NA
	1 ≤ T <2	NA	29,109/54,401 (53.51)	
	2 ≤ T <3	NA	7,359/12,289 (59.88)	
	3 ≤ T <4	NA	2,218/3,670 (60.44)	
	T≥4	NA	1,222/2,057 (59.41)	

*NO, Number; RMB, Renminbi (Yuan); T, Time; VI, Visual impairment*.

The overall prevalence rate of VI for all age groups between 6 and 18 years was 57.93% (52.55 to 63.30%), and the prevalence of VI before and during the COVID-19 pandemic appear to rise gradually from 53.48% (95%CI, 53.35 to 53.61) to 54.65% (95%CI, 54.51 to 54.78) (*P* < 0.001). The prevalence was also increased progressively for all ages from 53.68 to 54.90%. Highly statistically differences were detected at all age groups from 2019 to 2020 (*P* < 0.01) (Shown in Table 2).

**Table 2:** Prevalence of VI before and during COVID-pandemic among students aged 6 to 18 years.

**Age**	**Visual impairment Prevalence, No (%)**						***P*-value**
	**2019**			**2020**			
	**Total**	**Boys**	**Girls**	**Total**	**Boys**	**Girls**	
6	47	10 (43.48)	13 (56.52)	1,110	272 (50.09)	271 (49.91)	0.9982
7	61,252	11,321 (51.22)	10,782 (48.78)	28,220	5,301 (53.02)	4,698 (46.98)	0.0584
8	64,816	10,893 (51.75)	10,157 (48.25)	58,035	10,041 (53.61)	8,690 (46.39)	0.4518
9	60,685	10,842 (51.39)	10,254 (48.61)	58,194	10,662 (52.91)	9,489 (47.09)	0.6228
10	57,635	12,000 (49.77)	12,109 (50.23)	53,932	11,443 (51.50)	10,775 (48.50)	0.0317
11	56,761	13,950 (48.80)	14,636 (51.20)	50,050	12,274 (49.85)	12,348 (50.15)	0.0001
12	51,597	14,809 (49.36)	15,193 (50.64)	49,295	14,122 (49.44)	14,444 (50.56)	0.5247
13	44,068	14,572 (49.74)	14,722 (50.26)	46,363	15,302 (50.50)	14,999 (49.50)	0.0004
14	40,810	14,740 (49.84)	14,834 (50.16)	39,449	14,359 (50.67)	13,981 (49.33)	0.0472
15	38,095	14,932 (51.52)	14,051 (48.48)	39,000	15,190 (50.86)	14,679 (49.14)	0.0982
16	28,969	11,341 (49.26)	11,682 (50.74)	31,391	12,517 (50.80)	12,123 (49.20)	0.0031
17	26,644	10,702 (49.47)	10,930 (50.53)	24,137	9,590 (49.64)	9,729 (50.36)	0.0011
18	16,485	6,746 (49.87)	6,780 (50.13)	18,195	7,504 (51.78)	6,987 (48.22)	0.0000
Total	547,864	146,858 (50.12)	146,143 (49.88)	497,371	138,577 (50.99)	133,213 (49.01)	0.0000

*NO, Number; VI, Visual impairment*.

For students who went through both 2019 and 2020 examinations, a total of 153,205 were identified, 14.3% (21,887) students developed to VI during the COVID-19 pandemic while 33.7% (51,622) of them with normal vision in both years, and 44.3% (67,930) of them got persistently VI. The analysis showed that the VI incidence in Type 1 students (2019 without VI while 2020 with VI) was significantly increased with their total screen-based time (*P* < 0.001) (shown in Supplementary Table 3). Based on the multiple comparisons analysis, a similar trend was found that each type of VI change was influenced by the screen-based time (shown in Supplementary Table 4).

**Figure 1:**
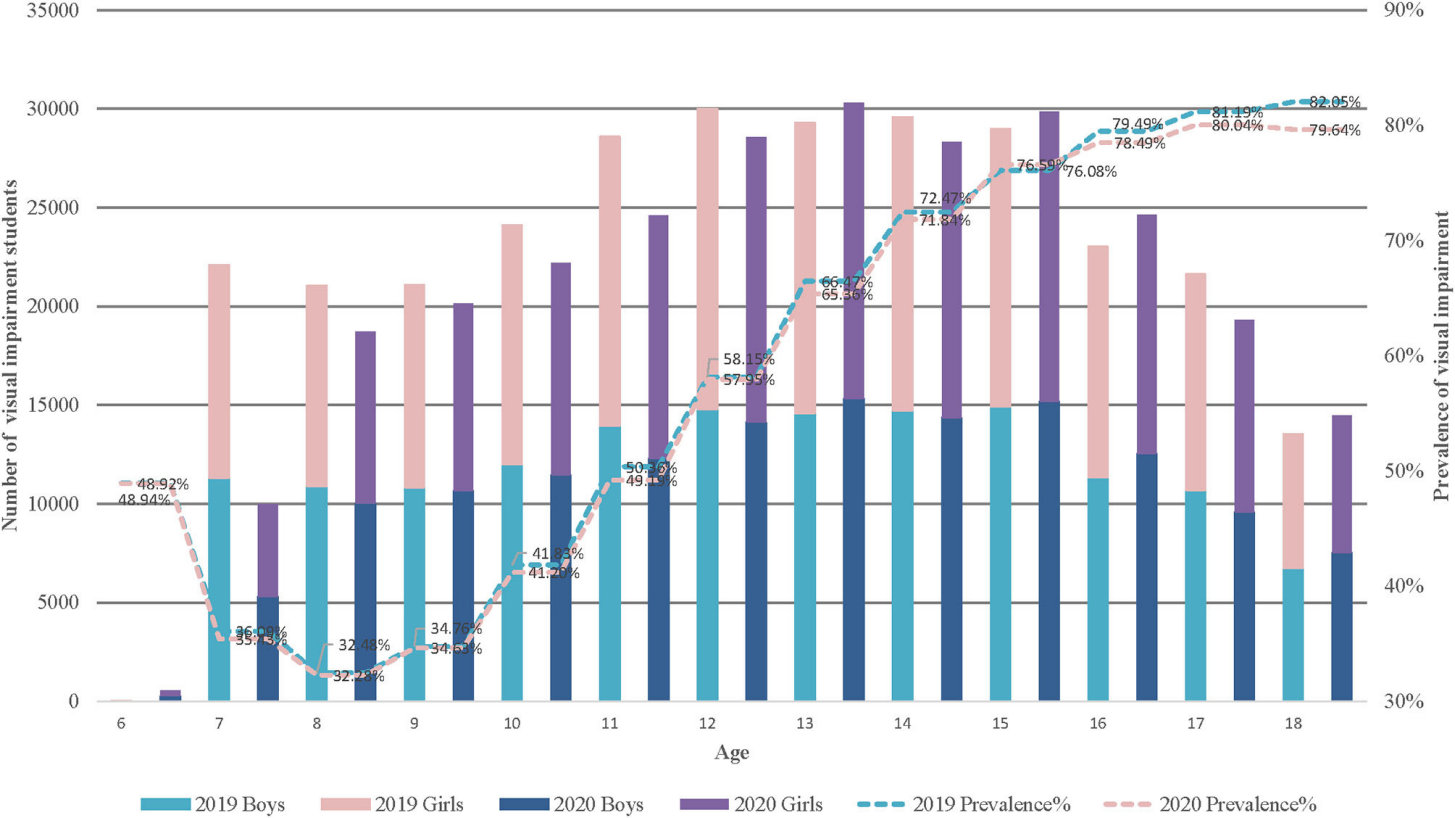
Prevalence of VI before and during COVID-pandemic among students aged 6 to 18 years. VI, Visual impairment.

The prevalence of VI actually showed an increased tendency with students' age and reaching the highest level in the 17 [(2019: 81.19%, 95%CI, 80.72 to 81.66) (2020: 80.04%, 95%CI, 79.53 to 80.54), *P* < 0.001] and 18 [(2019: 82.05%, 95%CI, 81.46 to 82.64) (2020: 79.64%, 95%CI, 79.06 to 80.23), *P* < 0.001] age groups. Similar finding was noticed that the high-school students [(2019: VI students = 58,359, 80.79%) (2020: VI students = 50,827, 79.42%), *P* < 0.001] obtain the highest prevalence according to the school stage. Rapid growth was detected between 9 [(2019: 34.76%, 95%CI, 34.38 to 35.14) (2020: 34.63%, 95%CI, 34.24 to 35.01)] (*P* = 0.62) and 14 [(2019: 72.47%, 95%CI, 72.03 to 72.90) (2020: 71.84%, 95%CI, 71.40 to 72.28)] (*P* < 0.05) years old. Prevalence levels for children aged from 6 to 12 were 48.94% (95%CI, 34.64 to 63.23), 36.09% (95%CI, 35.71 to 36.47), 32.48% (95%CI, 32.12 to 32.84), 34.76% (95%CI, 34.38 to 35.14), 41.83% (95%CI, 41.31 to 42.23), 50.36% (95%CI, 49.95 to 50.77), and 58.15% (95%CI, 57.72 to 58.57) in 2019 [2020: (48.92%, 95%CI, 45.98 to 51.86) (35.43%, 95%CI, 34.87 to 35.99) (32.28%, 95%CI, 31.89 to 32.66) (34.63%, 95%CI, 34.24 to 35.01) (41.20%, 95%CI, 40.78 to 41.61) (49.19%, 95%CI, 48.76 to 49.63), and (57.95%, 95%CI, 57.51 to 58.38)], respectively. For adolescent students aged from 13 to 18, corresponding value was 66.47% (95%CI, 66.03 to 66.92), 72.47% (95%CI, 72.03 to 72.90), 76.08% (95%CI, 75.65 to 76.51), 79.47% (95%CI, 79.01 to 79.94), 81.19% (95%CI, 80.72 to 81.66) and 82.05% (95%CI, 81.46 to 82.64) in 2019 [2020: (65.36%, 95%CI, 64.92 to 65.79) (71.84%, 95%CI, 71.40 to 72.28) (76.59%, 95%CI, 76.17 to 77.01) (78.49%, 95%CI, 78.04 to 78.95) (80.04%, 95%CI, 79.53 to 80.54) and (79.64%, 95%CI, 79.06 to 80.23)], respectively. Compared with girls, boys are more susceptible to develop VI both before (2019: boys, 50.12%; girls, 49.88%) and during the pandemic (2020:boys, 50.99%; girls, 49.01%), especially the students during the elementary school period. Moreover, we observed some significantly statistical difference at different stages of age between 2019 and 2020 years, especially the senior students (shown in Tables 2, 3 and Figure 1).

**Table 3:**
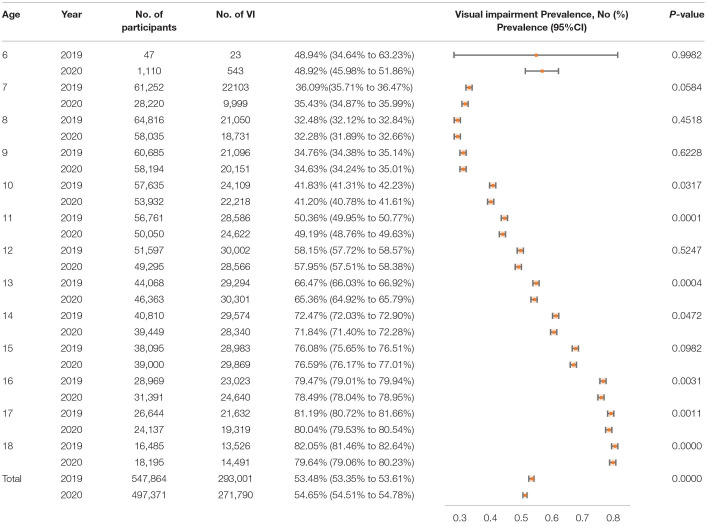
Prevalence of VI before and during COVID-pandemic among students aged 6 to 18 years.

*CI, Confidence interval; NO, Number; VI, Visual impairment*.

**Figure 2:**
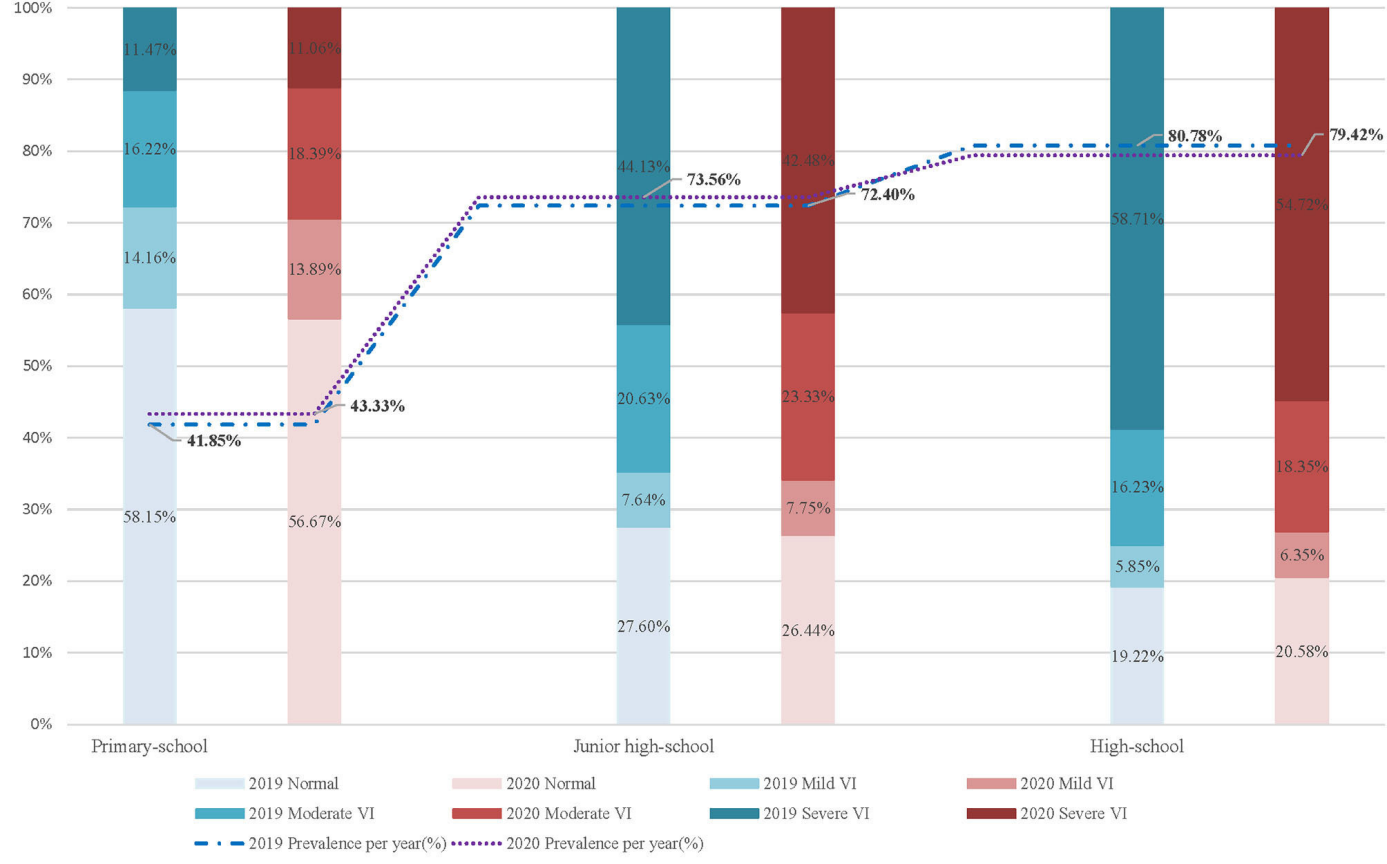
Prevalence of mild to severe VI before and during COVID-pandemic among school-aged students. NO, Number; PPY, Prevalence per year; VI, Visual impairment.

We found the VI prevalence before the pandemic of students at the primary school year varied from 14.16% [95%CI, 14.04 to 14.27 (*N* = 50,802)] in mild VI, 16.22% [95%CI, 16.10 to 16.34 (*N* = 58,220)] in moderate VI, 11.47% [95%CI, 11.37 to 11.58 (*N* = 41,165)] in severely VI, while the prevalence during the pandemic was 13.89 [95%CI, 13.77 to 14.01 (*N* = 44,914)], 18.39% [95%CI, 18.25 to 18.52 (*N* = 59,461)], and 11.06% [95%CI, 10.95 to 11.17 (*N* = 35,763)] for mild, moderate, and severe VI, respectively. At the junior high school level, the VI prevalence in 2019 was 7.64% [95%CI, 7.50 to 7.79 (*N* = 9,331)], 20.63% [95%CI, 20.41 to 20.86 (*N* = 25,191)], and 44.13% [95%CI, 43.85 to 44.41 (*N* = 53,872)] from mild to severe VI [2020: 7.75% (95%CI, 7.60 to 7.90, *N* = 9,025), 23.33% (95%CI, 23.09 to 23.58, *N* = 27,176), and 42.48% (95%CI, 42.20 to 42.76, *N* = 49,472)]. For students who entered in high school, the corresponding values in 2019 were 5.85% (95%CI, 5.68 to 6.02, *N* = 4,224), 16.23% (95%CI, 15.96 to 16.50, *N* = 11,722), and 58.71% (95%CI, 58.35 to 59.07, *N* = 42,413), respectively [2020: 6.35% (95%CI, 6.17 to 6.54, *N* = 4,065), 18.35% (95%CI, 18.05 to 18.65, *N* = 11,744), and 54.72% (95%CI, 54.33 to 55.10, *N* = 35,018)] (shown in Supplementary Table 1 and Figure 2).

## Discussion

Our study clarified the prevalence into age and school stage group based on pupil population aged 6 to 18 in Guangzhou, China. The increased prevalence of VI among all stages of students from 2019 (53.68%) to 2020 (54.90%) was still concerning.

The previous study has predicted that individuals with myopia will reach 4,758 million globally in 2050, which means nearly half of the world population will suffer from myopia (49.8%) (17). Severe deterioration of vision loss among people with myopia is commonly preceded by a preclinical phase representing the transitional adulthood stage between normal eyesight and myopia, namely, VI, but often ignored during the period of the student. Finding from Global Burden of Disease (GBD) indicated the individual suffered from moderate VI was increased by 133.67% million in China from 1990 to 2019 (18). The number of boys aged from 4 to 15 years with moderate VI was 0.75 million, while girls was 0.71 million in China in 2019 (19). During the COVID-19 pandemic, pandemic-related service closures, pressures, and public health policy have substantially increased the frequency for individuals to stay indoors whenever possible, which may have directly contributed to prolonging the time on sedentary behavior (SB), especially screen-based time. A similar situation is more pronounced for students as completion of academic requirements must be through the electronic instrument with telecommunication technology. For our surveyed students, daily homework was offered, of which primary students average 131.42 min while 149.17 and 156.45 min for junior high and high school students, respectively. The result refers to the time on screen-based behavior and also appears to be significantly prevalent in high consumption of screen-based behavior students [(screen-based time >4 h/d, 64.83%, 2019; 66.59%, 2020] compared with those spend less time on screen-based behavior [(screen-based time <1 h/d, 49.17%, 2019; 49.26%, 2020]. Such a general phenomenon can directly account for the difference in outdoor time between students who obtained more time outdoor [(outdoor time >2 h/d, 49.45%, 2019; 50.84%, 2020] and students who obtained less time [(outdoor time <1 h/d, 52.74%, 2019; 53.03%, 2020] when even the outdoor time was restricted due to the COVID-19 epidemic. Such considerable changes in VI did not occur in any other period, mainly due to home confinement in 2020. China is facing a serious and urgent public health concern of VI and much of the world, in all likelihood, would be encountering the same challenge.

Based on our finding, the VI prevalence for students aged 6 to 18 years old would be estimated to be approximately [53.48% (95%CI, 53.35 to 53.61)] and [54.65% (95%CI, 54.51 to 54.78)] in 2019 and 2020, respectively, which implied that every two students would develop VI. However, most interesting was the considerable increase in prevalence detected among students aged 9 to 16 years, which rises by 44.71% in 2020. For 17 and 18 year-old students, the shift was not conspicuous, but the overall VI prevalence for these age groups was already so serious that around four-fifths of students developed VI and the majority of them were involved in severe VI. One study has reported an evaluated substantial increase in the prevalence of myopia among school-aged children that the prevalence of myopia seems proximately 3 times higher in 2020 confinement compared with 2019 year for 6-year-old children while 2 times higher for children aged 7 years and 1.4 times for 8 years (7). Nevertheless, a significant VI shift was not observed among the older age groups (9 to 14 years), despite the older students encountering more tremendous academic pressure on online educational programs than younger students. Our finding partly agreed with the recent finding regarding myopia that the myopic prevalence among students aged from 6 to 8 appear to have experienced a period of rapid growth while 9 to 13 age groups were not seeing a substantial increase in myopia (7). However, with large scale-based sample size, our study has failed to obtain a significant finding that COVID-19-related restriction is associated with a rising but unobvious VI trend which was inconsistent with the previous publication and indicated a significant myopic shift after the COVID-19 home confinement. Several reasons may be explained. First, our study focuses on students aged from 6 to 18 while the former study selected children aged from 6 to 13. As we mentioned above, the younger students were more likely to suffer from VI due to the plasticity of their bodies. Second, there is substantial variation in study design, especially the measurement process of VI. Guangzhou comprises 1.8 million students that more than 200 professional medical institutions examined with around 6,000 medical physicians during September and October. In order to complete all the basic medical examinations, the quality during the vision screening process is hard to control, resulting in part of students with VI but not being detected. The insignificant VI trend is also probably due to the Guangzhou government's timely introduction of regional health policy to respond to the potential negative effects on students' health of the COVID-19 pandemic. As an example, based on the “student-school-family” social-ecological model, the Guangzhou Student Physical Health Platform provides ambulatory monitoring of health information for an individual student during the home-confinement period, which can generate an effective and timely personalized health prescription to manage an individual who is at potential risk for developing VI. Nevertheless, several COVID-19-related findings have been detected. The VI prevalence increased with the SB time, whether in entertainment or study-related time. Furthermore, based on the separate analysis conducted by including the students in both the 2019 and 2020 examinations, we found that the VI incidence (14.3%) was higher than the global myopic incidence (11.7%) during the COVID-19 pandemic has issued by China's Ministry of Education (8). Based on the COVID-19 related factors, our finding suggested the VI incidence was significantly increased with their total screen-based time among students without VI in 2019 while 2020 with VI (*P* < 0.001), which means that SB time increased during home confinement may develop to VI. On the other hand, we found that the prevalence of VI among high-school students declined 2 years in succession. It actually seemed less likely that the expeditious progression on VI in younger students arose from the overloaded burden on screen-based tasks since the learning assignments via the internet for them were less than older age students. In this view, the environmental change resulting from the COVID-19 pandemic might be the major reason for contributing the rapid progression in younger students due to they were more physically delicate and unstable for the response to the environment changing than older students. We may pose a hypothesis that younger students are more sensitive and fragility affected by the environmental factors than older students. It is reasonable to conclude that those more-influenced younger students, especially preschool children, were involved in a crucial VI development period, a time of high plasticity (20). Such age-shift is of high clinical relevance to the student population that final refractive error in adulthood was previously found to be related to a younger age of onset of VI. Therefore, enforcing prevention and control of VI in students would get more arduous during the exceptional time of environment change as they get older. On the whole, findings after the COVID-19 lockdown indicated that VI progression is associated with growth spurts in students' early stage while senior students' VI prevalence due to the plasticity is relatively low.

Additionally, although existing evidence suggested that the progression of prevalence on older age students was slower than younger students, the prevalence of VI on junior high-school [(2019:72.40%) (2020:73.56%)] and high-school [(2019:80.79%) (2020:79.42%)] students were ~1.7 and 2 times higher than primary school students [(2019:41.85%) (2020:43.34%)], respectively. And it is worth noting that two-thirds or more of senior students with VI mainly suffered from severe VI [(2019:58.71%) (2020:54.72%)]. The grade of VI was seemly downgraded as students get older. The leading causes of these changes were the continuously increased burden on enrollment pressure or unhealthy lifestyle during the COVID-19 pandemic, such as high frequency and close-up usage of students' eyes especially escalating amounts of time exposure to screens, and directly discontinuation of all vigorous outdoors activities due to classroom learning being replaced by home-based online learning. Furthermore, despite the fact that the reasons behind this shift are not evident, we found a difference between gender that appeared near the age from 6 to 10 years in the 2020 pandemic, that boys tended to have higher prevalence of VI than girls. However, girls seemed to commonly obtain the faster development than boys (21, 22) even as significantly unmodifiable risk factor for developing myopia (23), which probably was attributed to the advantages on their steeper corneas, steeper lens powers, and shorter axial length than boys (24, 25); moreover, there is the difference regarding puberty and change on estradiol level (26, 27), although relevant evidence is currently not solid yet.

Individual malnutrition, environmental population, daily lifestyle, as well as the inadequate access to quality visual care can considerably retard VI development (19, 28). The COVID-19 pandemic may indirectly promote the prevalence of VI. Indoors confinement may aggravate the burden of VI among students. The interactions on parents or other family members can directly affect the administration of use of students' eyes and they should limit screen time into a reasonable consumption, and increase the admissible outdoor activities while keeping safe social distance (29). Furthermore, relevant policies and management procedures need to be released and implemented for protecting the vision of juvenile students. For example, a notice on the management of mobile phones for primary and junior high students was issued by the China Ministry of Education, which requires the use of mobile phones among students should be supervised into routine management by schools and families collaboratively. Prevalence of myopia increased dramatically in east Asia, where the prevalence of myopia is 2 times higher than similarly aged white persons (30). The role of genetic predisposition to myopia or irreversible vision loss may in part explain the development of myopia over a short period (31), whereas the principal cause of myopia is widely considered to be driven by environmental factors including both the time spent outdoors and light environment, and the excessive use of eyes on screen-based behavior (32). Compared with European and American countries, young ages living in east Asia countries had a higher burden of so-called high-pressure academic task that may be a causative lifestyle change resulting from lack of time outdoors and excessive near-work activities (32). This phenomenon became more apparent as the pandemic intensified. The lockdown policy has been implemented more widely in China to prevent further spread of COVID-19, which also potentially contributes to the development of myopia since high-risk groups of students might have obtained fewer opportunistic screenings on their eyesight (33). Furthermore, indoor confinement restrictions on outdoor time and indoor space (most urban residents dwelling in multistory apartment buildings) increased screen time that may trigger the accommodative spasm (one of the vital factors in the refractive state during lockdown period) among students (29, 34). School-aged children and adolescents appear to be at higher risk for myopia development due to such populations being highly plastic, and control may be strongest during this age stage (20). Overall, the main current challenges against myopia during the COVID-19 pandemic included difficulty in seeing an ophthalmologist and lack of outdoor activity. In response to this dilemma, tele-ophthalmology screening programs is a potential technology that can substantially increase eyesight/VI screening rates and prevent myopia.

### Strengths

To the best of our knowledge, no prior study has reported the changing trends on VI status among students aged from 6 to 18 years before and during the COVID-19 pandemic based on Such a Large Series of Samples, Which Makes It Inaccessible to obtain effect estimates using a quantitative method. Furthermore, we randomly sampled half of the total sample size from each database to ensure the independence, reliability, and stability of our outputs. The larger the sample size, the better its broad representativeness, which indicates the accuracy of our estimation.

### Limitations

Despite the large sample size, we noted there were several limitations that should be acknowledged. First, there is a significant methodological limitation of our methodological dilemma to our measurement on VI. We screened the VI students according to the handbook of the Chinese National Student Constitution Survey Implementation Program. This measurement was distinguished from the Snellen chart approach recommended internationally. This difference would negatively affect our findings, and results may not extrapolate to the all-students group worldwide. Second, since GSSCH is a successive cross-sectional survey, some students were screened whether in the 2019 or 2020 pandemic. Such repeated measurement would repeat the VI records and lead to some biases to our analysis. Thus, we have addressed this issue by separate analysis of the students who were considered both in 2019 and 2020 examinations. Moreover, serval inherent bias such as information bias may be generated in large scale-based study, which should be acknowledged.

## Conclusions

Our finding suggests that the COVID-19 pandemic is associated with a rising but unobvious VI trend, which has provided a contemporary tocsin of the vulnerability related to visual disability among students before and during the COVID-19. Given the persistent, fast-increasing prevalence of VI from 6 to 14 years students and exceptionally high prevalence on high-school students, special concerns are still noticed for eliminating the potential impacts of the COVID-19 pandemic for VI among students.

## Data Availability Statement

The raw data supporting the conclusions of this article will be made available by the authors, without undue reservation.

## Ethics Statement

The studies involving human participants were reviewed and approved by the Ethics and Human Subject Committee of Sun Yat-sen University. The participants provided their written informed consent to participate in this study.

## Author Contributions

J-hL conducted the database search, screened and extracted data for the meta-analysis, prepared extracted data for the procedures, and had primary responsibility in writing this article. J-hL and Y-cC performed statistical analysis, interpretation of data, and drafted the initial manuscript. YZ, AK, NJ, SH, and S-xZ selected the articles, extracted the data, analyzed the data, and contributed to the discussion and editing. Y-jC supervised data collection and critically edited the final manuscript. All authors approved the final manuscript as submitted, agreed to be accountable for all aspects of the work, and read and approved the final manuscript.

## Funding

The work was supported by the National Natural Science Foundation of China (No. 81673193) and the Guangzhou Survey on Students' Constitution and Health (51000-73000368).

## Conflict of Interest

The authors declare that the research was conducted in the absence of any commercial or financial relationships that could be construed as a potential conflict of interest.

## Publisher's Note

All claims expressed in this article are solely those of the authors and do not necessarily represent those of their affiliated organizations, or those of the publisher, the editors and the reviewers. Any product that may be evaluated in this article, or claim that may be made by its manufacturer, is not guaranteed or endorsed by the publisher.
