# Supplementary material for: Prevalence of Visual Impairment Among Students Before and During the COVID-19 Pandemic, Findings From 1,057,061 Individuals in Guangzhou, Southern China
Source: Front Pediatr. 2022 Feb 11;9:813856. doi: 10.3389/fped.2021.813856 (PMC8875203; doi:10.3389/fped.2021.813856)
Supplement: Supplementary file 1 [file Data_Sheet_1.zip › Upload/Sup Table 1.DOCX]

**Supplementary Table.1 Prevalence of Mild to Severe VI before and during COVID-pandemic among School-Aged students**

| School stage | Year | Total | Visual impairment Prevalence, No(%) | | | | *P*-value |
| --- | --- | --- | --- | --- | --- | --- | --- |
|  |  |  | Total | Mild VI | Moderate VI | Severe VI |  |
|  |  |  | PPY, No(%) | PPY, No(%) | PPY, No(%) | PPY, No(%) |  |
| Primary-school | 2019 | 358,861 | 150,187, 41.85% (41.69% to 42.01%) | 50,802, 14.16% (14.04% to 14.27%) | 58,220, 16.22% (16.10% to 16.34%) | 41,165, 11.47% (11.37% to 11.58%) | 0.0001 |
|  | 2020 | 323,413 | 140,138, 43.33% (43.16% to 43.50%) | 44,914, 13.89% (13.77% to 14.01%) | 59,461, 18.39% (18.25% to 18.52%) | 35,763, 11.06% (10.95% to 11.17%) |  |
| Secondary-school | 2019 | 122,085 | 88,394,73.56% (73.31% to 73.82%) | 9,331, 7.64% (7.50% to 7.79%) | 25,191, 20.63% (20.41% to 20.86%) | 53,872, 44.13% (43.85% to 44.41%) | 0.0001 |
|  | 2020 | 116,461 | 85,673,80.78% (80.49% to 81.07%) | 9,025, 7.75% (7.60% to 7.90%) | 27,176, 23.33% (23.09% to 23.58%) | 49,472, 42.48% (42.20% to 42.76%) |  |
| High-school | 2019 | 72,241 | 58,359,79.42% (79.10% to 79.73%) | 4,224, 5.85% (5.68% to 6.02%) | 11,722, 16.23% (15.96% to 16.50%) | 42,413, 58.71% (58.35% to 59.07%) | 0.0001 |
|  | 2020 | 64,000 | 50,827,53.68% (53.55% to 53.81%) | 4,065, 6.35% (6.17% to 6.54%) | 11,744, 18.35% (18.05% to 18.65%) | 35,018, 54.72% (54.33% to 55.10%) |  |
| Total | 2019 | 553,187 | 296,940,54.90% (54.76% to 55.04%) | 64,357, 11.63% (11.55% to 11.72%) | 95,133, 17.20% (17.10% to 17.30%) | 137,450, 24.85% (24.73% to 24.96%) | 0.0001 |
|  | 2020 | 503,874 | 276,638,62.49% (53.17% to 71.81%) | 58,004, 11.51% (11.42% to 11.60%) | 98,381, 19.52% (19.42% to 19.63%) | 120,253, 23.87% (23.75% to 23.98%) |  |

**NO, Number; PPY, Prevalence per year; VI, Visual impairment.**
