# Supplementary material for: Prevalence of Visual Impairment Among Students Before and During the COVID-19 Pandemic, Findings From 1,057,061 Individuals in Guangzhou, Southern China
Source: Front Pediatr. 2022 Feb 11;9:813856. doi: 10.3389/fped.2021.813856 (PMC8875203; doi:10.3389/fped.2021.813856)
Supplement: Supplementary file 1 [file Data_Sheet_1.zip › Upload/Sup Table 2.DOCX]

**Supplementary Table. 2 Characteristics of VI change before and during COVID-19 pandemic among students who went through both in 2019 and 2020 examinations stratified by sociodemographic factors**

| **Parameters** | **Categories** | **VI change** | | | | | | | |
| --- | --- | --- | --- | --- | --- | --- | --- | --- | --- |
|  |  | **Type 1** | | **Type 2** | | **Type 3** | | **Type 4** | |
|  |  | **Non-VI in 2019** | **VI in 2020** | **Non-VI in 2019** | **Non-VI in 2020** | **VI in 2019** | **Non-VI in 2020** | **VI in 2019** | **VI in 2020** |
| Gender | Boys | 11,206(51.20) | 11,384(52.01) | 30,426(58.95) | 30,933(59.92) | 6,261(53.22) | 6,385(54.27) | 33,568(49.42) | 34,008(50.06) |
|  | girls | 10,680(48.80) | 10,503(47.99) | 21,190(41.05) | 20,689(40.08) | 5,504(46.78) | 5,381(45.73) | 34,360(50.58) | 33,922(49.94) |
| Age | Children | 7,614(80.82) | 10,961(73.68) | 19,522(86.33) | 29,132(81.60) | 4,227(82.14) | 6,081(77.63) | 14,583(51.47) | 17,511(43.47) |
|  | Adolescents | 1,807(19.18) | 3,915(26.32) | 3,090(13.67) | 6,569(18.40) | 919(17.86) | 1,752(22.37) | 13,750(48.53) | 22,775(56.53) |
| Grade | Primary-school | 17,689(80.82) | 15,612(71.33) | 43,680(84.62) | 40,339(78.14) | 9,290(78.96) | 8,547(72.64) | 33,032(48.63) | 25,511(37.55) |
|  | Secondary-school | 3,418(15.62) | 4,859(22.20) | 6,011(11.64) | 8,356(16.19) | 1,724(14.65) | 2,018(17.15) | 24,697(36.36) | 25,730(37.88) |
|  | High-school | 780(3.56) | 1,416(6.47) | 1,931(3.74) | 2,927(5.67) | 752(6.39) | 1,201(10.21) | 10,201(15.02) | 16,689(24.57) |
| Socioeconomic statues(monthly income/RMB) | ＜5000 | 4,031(42.79) | 6,126(45.70) | 10,024(44.33) | 15,578(48.43) | 2,122(41.24) | 3,209(45.43) | 11,869(41.89) | 15,976(44.04) |
|  | 5000 - 7999 | 1,930(20.49) | 3,106(23.17) | 4,660(20.61) | 7,326(22.78) | 1,092(21.22) | 1,646(23.30) | 5,836(20.60) | 8,514(23.47) |
|  | 8000 - 11999 | 1,151(12.22) | 1,852(13.81) | 2,555(11.30) | 4,114(12.79) | 612(11.89) | 904(12.80) | 3,523(12.43) | 5,234(14.43) |
|  | ≥12000 | 2,309(24.51) | 2,322(17.32) | 5,373(23.76) | 5,145(16.00) | 1,320(25.65) | 1,304(18.46) | 7,104(25.07) | 6,550(18.06) |
| Parental myopia | Yes | 4,485(47.61) | 7,377(49.59) | 9,278(41.03) | 14,902(41.74) | 2,362(45.90) | 3,636(46.42) | 15,096(53.28) | 22,202(55.11) |
|  | No | 4,936(52.39) | 7,499(50.41) | 13,334(58.97) | 20,799(58.26) | 2,784(54.10) | 4,197(53.58) | 13,236(46.72) | 18,084(44.89) |
| Total Outdoor time, h/d | T<1 | 3,751(39.82) | 6,648(44.69) | 9,022(39.90) | 15,526(43.49) | 2,094(40.69) | 3,543(45.23) | 12,004(42.37) | 18,307(45.44) |
|  | 1≤T<2 | 4,059(43.08) | 6,068(40.79) | 9,769(43.20) | 14,740(41.29) | 2,205(42.85) | 3,098(39.55) | 11,729(41.40) | 16,298(40.46) |
|  | T≥2 | 1,611(17.10) | 2,160(14.52) | 3,821(16.90) | 5,435(15.22) | 847(16.46) | 1,192(15.22) | 4,599(16.24) | 5,681(14.11) |
| Sunshine-related outdoor time, h/d | T<1 | 2,979(31.62) | 5,392(36.25) | 7,194(31.81) | 12,844(35.98) | 1,676(32.57) | 2,924(37.33) | 9,548(33.70) | 15,183(37.69) |
|  | 1≤T<2 | 4,099(43.51) | 6,332(42.57) | 9,820(43.43) | 15,091(42.27) | 2,223(43.20) | 3,237(41.33) | 12,164(42.93) | 16,850(41.83) |
|  | T≥2 | 2,343(24.87) | 3,152(21.19) | 5,598(24.76) | 7,766(21.75) | 1,247(24.23) | 1,672(21.35) | 6,620(23.36) | 8,253(20.48) |
| Total Screen-based time, h/d | T<1 | 5,732(62.45) | 10,413(70.24) | 12,937(58.81) | 23,415(65.87) | 3,187(63.59) | 5,388(69.02) | 19,180(69.29) | 30,548(76.06) |
|  | 1≤T<2 | 2,718(29.61) | 3,450(23.27) | 7,080(32.18) | 9,358(26.33) | 1,459(29.11) | 1,864(23.88) | 6,687(24.16) | 7,375(18.36) |
|  | 2≤T<3 | 556(6.06) | 695(4.69) | 1,487(6.76) | 2,024(5.69) | 286(5.71) | 418(5.35) | 1,320(4.77) | 1,597(3.98) |
|  | 3≤T<4 | 118(1.29) | 159(1.07) | 320(1.45) | 424(1.19) | 61(1.22) | 74(0.95) | 333(1.20) | 353(0.88) |
|  | T≥4 | 54(0.59) | 107(0.72) | 174(0.79) | 325(0.91) | 19(0.38) | 62(0.79) | 161(0.58) | 290(0.72) |
| Study-related screen-based time, h/d | T<1 | 337(3.62) | 575(3.88) | 796(3.57) | 1,584(4.46) | 179(3.52) | 316(4.05) | 998(3.57) | 1,793(4.46) |
|  | 1≤T<2 | 2,780(29.83) | 3,998(27.00) | 6,971(31.22) | 10,423(29.32) | 1,514(29.77) | 2,050(26.28) | 6,556(23.42) | 8,964(22.32) |
|  | 2≤T<3 | 3,666(39.33) | 5,557(37.53) | 8,907(39.89) | 13,520(38.03) | 1,972(38.77) | 2,900(37.18) | 10,253(36.63) | 13,953(34.75) |
|  | 3≤T<4 | 1,824(19.57) | 3,292(22.23) | 4,154(18.60) | 7,120(20.03) | 1,009(19.84) | 1,753(22.47) | 6,880(24.58) | 10,025(24.96) |
|  | T≥4 | 713(7.65) | 1,385(9.35) | 1,500(6.72) | 2,908(8.18) | 412(8.10) | 781(10.01) | 3,307(11.81) | 5,422(13.50) |
| Entertainment-related screen-based time, h/d | T<1 | NA | 8,749(59.02) | NA | 21,746(61.12) | NA | 4,887(62.61) | NA | 21,441(53.37) |
|  | 1≤T<2 | NA | 4,022(27.13) | NA | 9,652(27.13) | NA | 2,030(26.01) | NA | 10,864(27.04) |
|  | 2≤T<3 | NA | 1,248(8.42) | NA | 2,548(7.16) | NA | 527(6.75) | NA | 4,450(11.08) |
|  | 3≤T<4 | NA | 433(2.92) | NA | 808(2.27) | NA | 190(2.43) | NA | 1,666(4.15) |
|  | T≥4 | NA | 371(2.50) | NA | 827(2.32) | NA | 172(2.20) | NA | 1,750(4.36) |

**VI, visual impairment; Type 1, 2019 without VI while 2020 with VI; Type 2, 2019 without VI while 2020 without VI; Type 3, 2019 with VI while 2020 without VI; Type 4, 2019 with VI while 2020 with VI.**
