# Supplementary material for: Prevalence of Visual Impairment Among Students Before and During the COVID-19 Pandemic, Findings From 1,057,061 Individuals in Guangzhou, Southern China
Source: Front Pediatr. 2022 Feb 11;9:813856. doi: 10.3389/fped.2021.813856 (PMC8875203; doi:10.3389/fped.2021.813856)
Supplement: Supplementary file 1 [file Data_Sheet_1.zip › Upload/Sup Table 3.DOCX]

**Supplementary Table.3 Differences of time change among students without VI in 2019 while 2020 with VI stratified by physical activity and sedentary behavior factors**

| **Parameters** | **Categories for time change, h/d** | **Non-VI in 2019** | **VI in 2020** | ***P*-Value** |
| --- | --- | --- | --- | --- |
| Change of Total Outdoor time | -3 | 285(1.28) | 365(1.33) | 0.387 |
|  | -2 | 1,140(5.12) | 1,483(5.40) |  |
|  | -1 | 4,954(22.26) | 6,056(22.05) |  |
|  | 0 | 10,383(46.66) | 12,800(46.61) |  |
|  | 1 | 4,214(18.94) | 5,244(19.10) |  |
|  | 2 | 1,007(4.53) | 1,170(4.26) |  |
|  | 3 | 271(1.22) | 341(1.24) |  |
| Change of Sunshine-related outdoor time | -3 | 334(1.50) | 446(1.62) | 0.464 |
|  | -2 | 1,362(6.12) | 1,803(6.57) |  |
|  | -1 | 5,076(22.81) | 6,201(22.58) |  |
|  | 0 | 9,939(44.66) | 12,154(44.26) |  |
|  | 1 | 4,154(18.67) | 5,116(18.63) |  |
|  | 2 | 1,122(5.04) | 1,358(4.95) |  |
|  | 3 | 267(1.20) | 381(1.39) |  |
| Change of Total Screen-based time | -4 | 49(0.23) | 124(0.47) | 0.000 |
|  | -3 | 114(0.53) | 251(0.94) |  |
|  | -2 | 396(1.84) | 824(3.09) |  |
|  | -1 | 3,028(14.04) | 3,838(14.41) |  |
|  | 0 | 13,309(61.72) | 14,630(54.93) |  |
|  | 1 | 3,455(16.02) | 4,731(17.76) |  |
|  | 2 | 772(3.58) | 1,439(5.40) |  |
|  | 3 | 299(1.39) | 553(2.08) |  |
|  | 4 | 142(0.66) | 242(0.91) |  |
| Change of Study-related screen-based time | -4 | 28(0.13) | 50(0.18) | 0.861 |
|  | -3 | 227(1.04) | 400(1.48) |  |
|  | -2 | 1,099(5.02) | 1,601(5.92) |  |
|  | -1 | 4,507(20.57) | 5,293(19.56) |  |
|  | 0 | 9,216(42.07) | 10,902(40.29) |  |
|  | 1 | 5,125(23.39) | 6,578(24.31) |  |
|  | 2 | 1,407(6.42) | 1,795(6.63) |  |
|  | 3 | 271(1.24) | 393(1.45) |  |
|  | 4 | 28(0.13) | 47(0.17) |  |

**VI, visual impairment; Type of independent variables are categorized by the time change from 2019 to 2020.**
