# Supplementary material for: Prevalence of Visual Impairment Among Students Before and During the COVID-19 Pandemic, Findings From 1,057,061 Individuals in Guangzhou, Southern China
Source: Front Pediatr. 2022 Feb 11;9:813856. doi: 10.3389/fped.2021.813856 (PMC8875203; doi:10.3389/fped.2021.813856)
Supplement: Supplementary file 1 [file Data_Sheet_1.zip › Upload/Sup Table 4.DOCX]

**Supplementary Table.4 Characteristics of VI change before and during COVID-19 pandemic among students who went through both in 2019 and 2020 examinations stratified by physical activity and sedentary behavior factors**

| **Parameters** | **Categories for time change, h/d** | **VI change** | | | | |
| --- | --- | --- | --- | --- | --- | --- |
|  |  | **VI change type 1** | **VI change type 2** | **VI change type 3** | **VI change type 4** | ***P*-Value** |
| Change of Total Outdoor time | -3 | 102(1.36) | 230(1.26) | 55(1.36) | 263(1.32) | 0.614 |
|  | -2 | 402(5.36) | 940(5.16) | 200(4.95) | 1,081(5.42) |  |
|  | -1 | 1,672(22.29) | 4,060(22.29) | 894(22.15) | 4,384(21.97) |  |
|  | 0 | 3,512(46.83) | 8,476(46.53) | 1,907(47.24) | 9,288(46.54) |  |
|  | 1 | 1,421(18.95) | 3,461(19.00) | 753(18.65) | 3,823(19.15) |  |
|  | 2 | 303(4.04) | 830(4.56) | 177(4.38) | 867(4.34) |  |
|  | 3 | 88(1.17) | 220(1.21) | 51(1.26) | 253(1.27) |  |
| Change of Sunshine-related outdoor time | -3 | 121(1.61) | 268(1.47) | 66(1.63) | 325(1.63) | 0.392 |
|  | -2 | 491(6.55) | 1,132(6.21) | 230(5.70) | 1,312(6.57) |  |
|  | -1 | 1,661(22.15) | 4,137(22.71) | 939(23.26) | 4,540(22.75) |  |
|  | 0 | 3,333(44.44) | 8,142(44.69) | 1,797(44.51) | 8,821(44.20) |  |
|  | 1 | 1,434(19.12) | 3,393(18.63) | 761(18.85) | 3,682(18.45) |  |
|  | 2 | 357(4.76) | 922(5.06) | 200(4.95) | 1,001(5.02) |  |
|  | 3 | 103(1.37) | 223(1.22) | 44(1.09) | 278(1.39) |  |
| Change of Total Screen-based time | -4 | 31(0.43) | 42(0.24) | 7(0.18) | 93(0.48) | 0.000 |
|  | -3 | 54(0.74) | 94(0.53) | 20(0.51) | 197(1.02) |  |
|  | -2 | 159(2.19) | 326(1.85) | 70(1.79) | 665(3.43) |  |
|  | -1 | 1,019(14.04) | 2,509(14.22) | 519(13.26) | 2,819(14.55) |  |
|  | 0 | 4,318(59.50) | 10,864(61.55) | 2,445(62.47) | 10,312(53.22) |  |
|  | 1 | 1,208(16.65) | 2,808(15.91) | 647(16.53) | 3,523(18.18) |  |
|  | 2 | 310(4.27) | 656(3.72) | 116(2.96) | 1,129(5.83) |  |
|  | 3 | 114(1.57) | 234(1.33) | 65(1.66) | 439(2.27) |  |
|  | 4 | 44(0.61) | 117(0.66) | 25(0.64) | 198(1.02) |  |
| Change of Study-related screen-based time | -4 | 7(0.09) | 26(0.14) | 2(0.05) | 43(0.22) | 0.074 |
|  | -3 | 90(1.22) | 186(1.04) | 41(1.03) | 310(1.58) |  |
|  | -2 | 391(5.29) | 892(4.97) | 207(5.21) | 1,210(6.15) |  |
|  | -1 | 1,459(19.75) | 3,698(20.62) | 809(20.36) | 3,834(19.49) |  |
|  | 0 | 3,014(40.80) | 7,584(42.29) | 1,632(41.07) | 7,888(40.10) |  |
|  | 1 | 1,831(24.78) | 4,154(23.16) | 971(24.43) | 4,747(24.13) |  |
|  | 2 | 474(6.42) | 1,143(6.37) | 264(6.64) | 1,321(6.72) |  |
|  | 3 | 107(1.45) | 227(1.27) | 44(1.11) | 286(1.45) |  |
|  | 4 | 15(0.20) | 24(0.13) | 4(0.10) | 32(0.16) |  |
