# Supplementary material for: Prevalence of Visual Impairment Among Students Before and During the COVID-19 Pandemic, Findings From 1,057,061 Individuals in Guangzhou, Southern China
Source: Front Pediatr. 2022 Feb 11;9:813856. doi: 10.3389/fped.2021.813856 (PMC8875203; doi:10.3389/fped.2021.813856)
Supplement: Supplementary file 1 [file Data_Sheet_1.zip › Upload/Sup Table 4_1.DOCX]

**Supplementary Table.4 Characteristics of VI change before and during COVID-19 pandemic among involved students**

| Parameter | Categories | No, of participants/% | | *P*-Value |  |
| --- | --- | --- | --- | --- | --- |
|  |  | 2019 | 2020 |  |  |
| Gender | Boys | 296,757(53.6) | 283,543(54.5) | ＜0.001 |  |
|  | girls | 256,567(46.4) | 236,910(45.5) |  |  |
| Age | Children | 141,584(70.9) | 231.024(69.7) | ＜0.001 |  |
|  | Adolescents | 58,153(29.1) | 100,602(30.3) |  |  |
| Grade | Primary-school | 115,032(63.8) | 207,443(62.6) | 0.810 |  |
|  | Secondary-school | 41,699(23.1) | 84,582(25.5) |  |  |
|  | High-school | 23,649(13.1) | 39,601(11.9) |  |  |
| Socioeconomic statues(monthly income/RMB) | ＜5000 | 80,889(40.4) | 136,327(45.7) | ＜0.001 |  |
|  |  |  |  |  |  |
|  | 5000 - 7999 | 41,574(20.8) | 68,984(23.1) |  |  |
|  | 8000 - 11999 | 25,047(12.5) | 40,980(13.7) |  |  |
|  | ≥12000 | 52,495(26.2) | 51,955(17.4) |  |  |
|  |  |  |  |  |  |
| Parental myopia | Yes | 97,601(48.8) | 162,318(48.9) | 0.3 |  |
|  | No | 102,404(51.2) | 169,308(51.1) |  |  |
| Total Outdoor time, h/d | T<1 | 82,625(41.3) | 147,247(44.4) | ＜0.001 |  |
|  | 1≤T<2 | 84,612(42.3) | 135,906(41.0) |  |  |
|  | T≥2 | 32,768(16.4) | 48,472(14.6) |  |  |
| Sunshine-related outdoor time, h/d | T<1 | 66,114(33.1) | 121,679(36.7) | ＜0.001 |  |
|  | 1≤T<2 | 86,514(43.3) | 139,646(42.1) |  |  |
|  | T≥2 | 47,377(23.7) | 70,300(21.2) |  |  |
| Total Screen-based time, h/d | T<1 | 122,962(63.2) | 197,373(59.7) | ＜0.001 |  |
|  | 1≤T<2 | 50,705(26.1) | 86,803(26.3) |  |  |
|  | 2≤T<3 | 12,924(6.6) | 27,168(8.2) |  |  |
|  | 3≤T<4 | 4,251(2.2) | 9,473(2.9) |  |  |
|  | T≥4 | 3,603(1.9) | 9,665(2.9) |  |  |
| Study-related screen-based time, h/d | T<1 | 7,084(3.6) | 14,420(4.4) | 0.093 |  |
|  | 1≤T<2 | 53,294(27.0) | 88,627(26.8) |  |  |
|  | 2≤T<3 | 75,448(38.2) | 122,683(37.1) |  |  |
|  | 3≤T<4 | 43,217(21.9) | 71,692(21.7) |  |  |
|  | T≥4 | 18,575(9.4) | 32,870(10.0) |  |  |
| Entertainment-related screen-based time, h/d | T<1 | NA | 231,646(70.1) |  |  |
|  | 1≤T<2 | NA | 76,526(23.2) |  |  |
|  | 2≤T<3 | NA | 16,218(4.9) |  |  |
|  | 3≤T<4 | NA | 3,348(1.0) |  |  |
|  | T≥4 | NA | 2,672(0.8) |  |  |

**NO, Number; RMB, Renminbi(Yuan); T, Time; VI, Visual impairment.**
